# The Role of Immunobiotics and Postbiotics in the Recovery of Immune Cell Populations From Respiratory Mucosa of Malnourished Hosts: Effect on the Resistance Against Respiratory Infections

**DOI:** 10.3389/fnut.2021.704868

**Published:** 2021-08-12

**Authors:** Susana Salva, Yanina Kolling, Maximiliano Ivir, Florencia Gutiérrez, Susana Alvarez

**Affiliations:** ^1^Laboratory of Immunobiotechnology, Reference Centre for Lactobacilli Centro de Referencia para Lactobacilos-Consejo Nacional de Investigaciones Científicas y Técnicas (CERELA-CONICET), San Miguel de Tucuman, Argentina; ^2^Clinical Biochemistry I, Institute of Applied Biochemistry, National University of Tucuman, San Miguel de Tucuman, Argentina

**Keywords:** immunobiotic, postbiotic, malnutrition, respiratory infections, respiratory mucosal immunity

## Abstract

Malnutrition is associated with a state of secondary immunodeficiency, which is characterized by a worsening of the immune response against infectious agents. Despite important advances in vaccines and antibiotic therapies, the respiratory infections are among the leading causes of increased morbidity and mortality, especially in immunosuppressed hosts. In this review, we examine the interactions between immunobiotics-postbiotics and the immune cell populations of the respiratory mucosa. In addition, we discuss how this cross talk affects the maintenance of a normal generation of immune cells, that is crucial for the establishment of protective innate and adaptive immune responses. Particular attention will be given to the alterations in the development of phagocytic cells, T and B lymphocytes in bone marrow, spleen and thymus in immunosuppression state by protein deprivation. Furthermore, we describe our research that demonstrated that the effectiveness of immunobiotics nasal administration in accelerating the recovery of the respiratory immune response in malnourished hosts. Finally, we propose the peptidoglycan from the immunobiotic *Lactobacillus rhamnosus* CRL1505 as the key cellular component for the effects on mucosal immunity, which are unique and cannot be extrapolated to other *L. rhamnosus* or probiotic strains. In this way, we provide the scientific bases for its application as a mucosal adjuvant in health plans, mainly aimed to improve the immune response of immunocompromised hosts. The search for safe vaccine adjuvants that increase their effectiveness at the mucosal level is a problem of great scientific relevance today.

## Introduction

Malnutrition is a serious condition characterized by inadequate intake of both energy and macronutrients (carbohydrates, proteins, fats) as well as to micronutrient (minerals and vitamins) deficiency (1). For the WHO, malnutrition consists of both undernutrition and overweight and obesity, as well as diet-related non-communicable diseases (2). However, the European Society of Clinical Nutrition and Metabolism guidelines consider malnutrition and undernutrition as synonyms and define them as nutritional disorders (3). Given the lack of a global consensus on diagnostic criteria, along with new evidence supporting the influence of disease and inflammation on malnutrition, the Global Leadership Initiative on Malnutrition (GLIM) involved most nutrition societies in an effort to standardize the diagnosis of malnutrition in clinical settings (1, 3). GLIM proposes a three-step assessment: first, patients must be identified using a validated screening tool; second, malnutrition requires the presence of at least one phenotypic criterion and one etiological criterion; and finally, the severity is based on the threshold levels of the phenotypic criteria. Regarding the etiology, GLIM classified malnutrition caused by a chronic disease, distinguishing presence or absence of inflammation, by an acute inflammatory disease or by starvation (for socioeconomic or environmental causes that imply food shortages or hunger) (1, 3). While the number of overweight children around the world has remained stagnant for over a decade, 144 million children under the age of 5 were stunted and 47 million suffered from wasting in 2019 (4, 5). Furthermore, undernutrition is associated with 45% of deaths among children younger than 5 years old. This occurs mainly in low- and middle-income countries (4).

Before the Covid-19 pandemic, almost 690 million people were victims of chronic hunger. During 2020, the number of vulnerable children suffering from malnutrition was greater due to the deterioration in their diet quality and the repercussions of measures to contain the pandemic (6). The first measures used to prevent the COVID-19 transmission disrupted food systems, health and nutrition services, devastating livelihoods and threatening food security. Faced with this situation, UNICEF, the Food and Agricultural Organization, the World Food Program and the World Health Organization, issued a call to action, warning about the pandemic's potential to worsen the pre-existing crisis of malnutrition and tip an additional 6.7 million children over the edge to become wasted during the first year (7).

**Figure 1:**
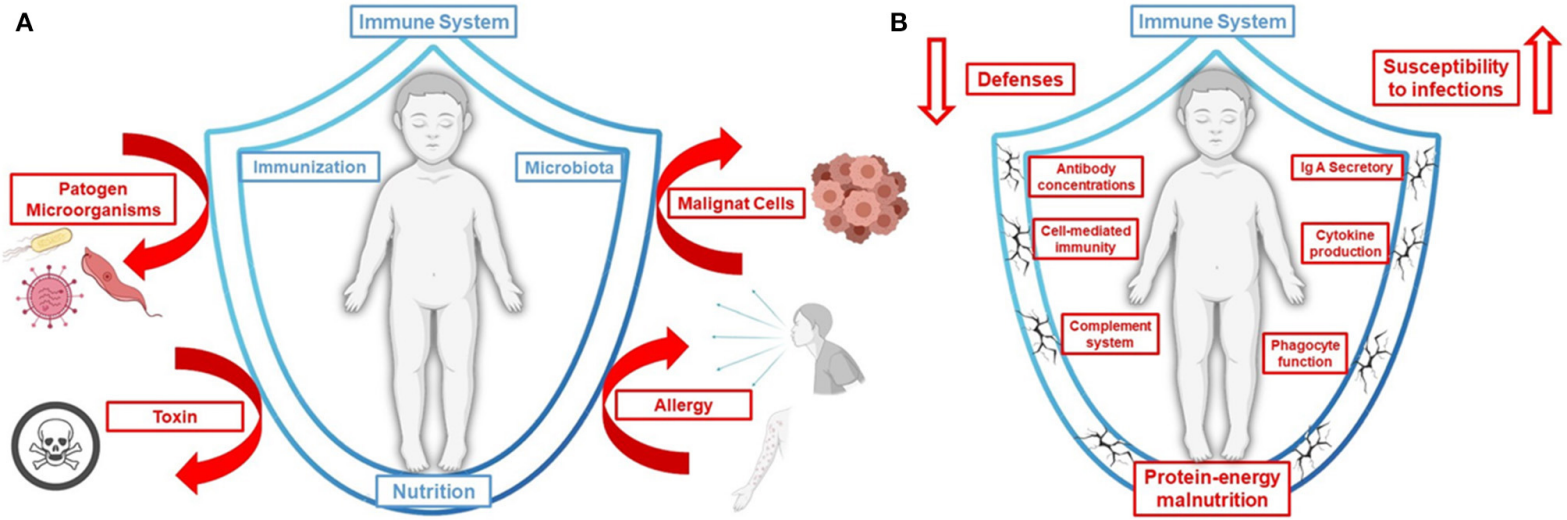
Simple view of immune system as a shield. **(A)** An adequate nutritional state, a balanced microbiota and a correct immunization guarantee the strengthening of our immune defenses. **(B)** Protein-energy malnutrition is associated with immune dysfunction and an increase of susceptibility to infectious diseases. In turn, immune responses to infectious diseases enhance nutrient requirements, reduce appetite, and impede the absorption of nutrients creating and perpetuating a vicious cycle (Figure created with Biorender.com).

The nutritional status affects all aspects of health, including normal growth and development, and immune response against diseases. Undernutrition is characterized by a cellular imbalance between nutrient/energy supply and the demands of the body's cells, leading to impaired immune system function among other alterations (Figure 1) (8). Recent research has emphasized a preponderant role of the nutritional status of the host in his/her resistance to infection and as a mediator of its effects (9, 10). Malnutrition, especially in children and the elderly, induces a higher risk of dying from common infections, and increases their frequency and severity, delaying recovery (7). The interaction between malnutrition and infection can create a life-threatening cycle of disease exacerbation and deteriorating nutritional status (Figure 1).

Despite important advances in vaccines and antibiotic therapies, the respiratory infections are among the leading causes of increased morbidity and mortality in immunosuppressed hosts. A clear example is observed in the alarming growth of epidemiological data of the current pandemic by SARS-CoV-2, where the lack of availability of vaccines or effective treatments has the world scientific community on edge. Thus, a healthy immune system is the most important weapon against this and other infections. Several clinical and animal model studies have demonstrated the ability of immunobiotics to beneficially modulate respiratory immunity (11–14). In this sense, our research group has shown that the mucosal administration (oral or nasal) of some inmunobiotic strains or their postbiotics (cell wall and peptidoglycan) can beneficially modulate respiratory immunity, improving the immune response against bacterial and viral infections in immunocompetent and immunocompromised hosts (15–20). In this way, we consider that immunobiotic bacteria can be used in therapies aimed at modulating the immunity of the respiratory mucosa, especially in populations at risk.

In this review, we examine the interactions between immunobiotics or postbiotics and the immune cell populations of the respiratory mucosa. In addition, we discuss how this cross talk affects the maintenance of a normal generation of immune cells which is crucial for the establishment of protective innate and adaptive immune responses. Furthermore, we describe the result that demonstrated the effectiveness of immunobiotics or their postbiotics nasal administration in accelerating the recovery of the respiratory immune response in malnourished hosts.

## Protein-Malnutrition Impairs the Respiratory Innate Immune Response Against Pneumococcal Infection

There is a growing appreciation of malnutrition as a set of overlapping comorbidities that are not well-known (21–23). Understanding the pathogenesis of undernutrition across the spectrum is essential to support current international goals with novel therapeutic approaches to improve nutrition, health, and well-being (7). Lack of protein is known to affect people more significantly at a very young age or much later in life. Older malnourished people are at risk of prolonged hospital stays, infections, impaired respiratory function, and death (24–26). Malnourished children by protein deprivation die mainly from common infections (23, 27), implying that mortality is related to underlying immunodeficiency, even in mild forms of undernutrition (28). Our approach is directed to childhood malnutrition that affects the development and performance of the immune system. Protein-deprived immune dysfunction involves innate and adaptive immunity and is therefore a key factor in the vicious cycle that leads to clinical malnutrition (Figure 1). In the last decades, high number of investigations about failures in innate and adaptive immunity in malnourished children (23, 27) as well as in experimental models (16, 29) have been systematically published. Such alterations affect the ability to respond especially against bacterial and viral respiratory pathogens. Respiratory infections are among the leading causes of morbidity and mortality in children, and are caused by both asymptomatically residing bacteria with pathogenic potential, and pathogenic bacterial species, such as *Streptococcus pneumoniae* (30, 31). This respiratory pathogen colonizes the nasopharynx asymptomatically in healthy humans with higher colonization rates in children. However, it causes otitis media in 50% of cases and is the most common cause of bacterial pneumonia in humans (32). *S. pneumoniae* can also induce invasive septicemia and meningitis with high mortality rates. In developed countries, rates of pneumococcal disease have dropped considerably in recent years due to vaccination. Nevertheless, it continues to be an important cause of morbidity and mortality seen globally in immunosuppressed patients and particularly causing about 810,000 deaths in children under 5 years of age (30, 31). Currently, COVID-19 co-infections are associated with a higher number of fatal cases compared to those with COVID-19 alone (33, 34). An adequate innate and adaptive immune response is essential to eradicate *S. penumoniae* from the host (16, 18). Several factors could be involved in the impairment of innate immune response against *S. pneumoniae* in protein-deprived malnourished hosts.

Alveolar macrophages are the first cells to be activated by pulmonary infection and their response is especially important in *S. pneumoniae* infection (18). Protein deprivation reduces the number of the two main populations of alveolar macrophages at steady state, the lung-resident alveolar macrophages with self-renewal capacity, and those derived from monocytes. Faced with a bacterial load that exceeds the ability of alveolar macrophages to phagocytize, they can coordinate a pro-inflammatory and antimicrobial local environment by recruiting additional phagocytes critical for bacterial clearance (35). However, protein-deprived malnourished mice have difficulty eradicating a lung pathogen because, on the one hand, their alveolar macrophages produce low levels of mediators necessary for recruitment, such as TNF-α, IL-1β and IL-6 at the local and systemic level. On the other hand, the populations of neutrophils, monocytes, macrophages, and dendritic cells (DC) are impaired by malnutrition (36–38). In addition, protein deprivation reduces the effectiveness of alveolar macrophages to coordinate an anti-inflammatory environment to facilitate lung tissue repair, preventing the resolution of inflammation and causing excessive tissue damage (19, 39, 40). Namely, IL-10 production during pneumococcal infection, a critical anti-inflammatory cytokine necessary to control excessive lung inflammation, is reduced by protein deprivation (19, 38, 40).

It is known that neutrophils are key cells during a respiratory infection (41) and malnutrition impairs steady-state and emergency granulopoiesis in mice (29, 38). Consequently, protein-deprived malnourished mice suffer from leukopenia and neutropenia, especially during pneumococcal infection, and show a reduced capacity to recruit neutrophils into infected lungs (19, 38), A reduced expression of CXCL12 is observed in bone marrow during a pneumococcal infection in malnourished mice by protein deprivation, possibly as a mechanism for the preservation of hematopoietic stem cells (29, 42). At the same time, the lack of increase in GM-CSF and IL-1 in bone marrow is responsible for a defective emergency granulopoiesis against the infectious challenge by not expanding the multipotent progenitors and common lymphoid and myeloid progenitors (29, 43, 44). In addition, protein deprivation can influence following events in neutrophil homeostasis such as their functionality. It was demonstrated that the protein deprivation induces an impairment of the myeloperoxidase activity and the phagocytic capacity of the cells of the broncho-alveolar lavage and blood in mice (19, 44). This impairment partly reflects the functional limitations of immature cells (44). Thus, malnutrition is responsible for the failure of pneumococcal clearance from the lungs, and for an unproductive inflammatory response.

## Immunobiotics and Postbiotics Accelerate the Respiratory Innate Immunity Recovery of Malnourished Hosts

There is recent evidence of the immunobiotics' use as dietary supplements to enhance immunity and resistance against infection in protein-deprived malnourished hosts. Most of the bibliography demonstrates the potential of oral administration of immunobiotics to beneficially modulate respiratory immunity by studies in animal models and clinical trials (16, 18, 29). In addition, nasal administration of immunobiotics has been proposed to preferentially induce systemic immunity and especially stimulate the tissue of the respiratory mucosa, which provides an advantage in protection against respiratory pathogens (18, 38, 45). The greater efficacy of the intranasal probiotic compared to the oral route could be due to the stronger stimulation of the immune cells of the airways in the nasal cavity and the upper respiratory tract. However, intranasal delivery of immunobiotics is poorly understood and warrants further studies. Several nasal immunobiotic treatments are known, mainly used to beneficially modulate the immune response in models of respiratory infection, allergy and chronic obstructive pulmonary disease, but very are not known in malnutrition models (45).

It is important to note that the administration of viable microorganisms could imply a risk to the health of immunosuppressed patients (46), consequently the use of postbiotics could be an interesting alternative to stimulate immunity (19, 47). In this sense, it is known that the host's response to an immunobiotic depends on the combination of the different bacterial molecules that can interact with the various receptors on the host cells (48). The cell wall, peptidoglycan, exopolysaccharides or secreted metabolites are most commonly molecules of immunobiotic bacteria that are associated with immunomodulatory effects and their beneficial impact on health (49–51). However, there is little literature about nasal administration of postbiotics.

Keeping these concepts in mind, we will now describe the most relevant findings of the beneficial effects of nasal administration of immunobiotics and postbiotics on the innate immune response in the context of malnutrition by protein deprivation. The nasal administration of immunobiotics *Lactobacillus casei* CRL431 or *Lactobacillus rhamnosus* CRL1505 during a mice repletion diet induces an increase in the number and activity of phagocytic cells of the respiratory mucosa before an infectious challenge (37, 40). In particular, *L. rhamnosus* CRL1505 is able to normalize the number of monocytes and alveolar macrophages in the lungs (37). CRL1505 strain is also able to up-regulate the expression of the MHC II activation marker in lung DCs and to improve CD11b^+^ DC population specially (37). Even more, the peptidoglycan obtained from *L. rhamnosus* CRL1505 (PG05) is able to increase the number and activation of alveolar macrophages isolated from bronchoalveolar lavage. These is determined by an increase in the expression of MHCII in CD11c^+^F4/80^+^ cells before an infectious challenge (19, 20). In line with these findings, the mice renourished with the nasally administered immunobiotic CRL1505 or its PG05 normalize the number of neutrophils and increase the positive peroxidase cells of peripheral blood (19, 20, 38). These effects are related to the acceleration of the recovery of the steady-state myelopoiesis affected by protein deprivation (38). Renutrition treatments supplemented nasally with *L. rhamnosus* CRL1505 and PG05 are effective in restoring bone marrow tissue architecture, increasing proliferating bone marrow cells, Gr-1^high^ mature myeloid cells, and neutrophils. Although the mechanisms are not known, it has been shown that peptidoglycan from the microbiota can be found in the neutrophil fraction in the bone marrow, and exert a physiological stimulation of steady-state myelopoiesis (28, 41). Our findings reinforce the idea that microbial products benefit the host by enhancing systemic innate immune function.

**Figure 2:**
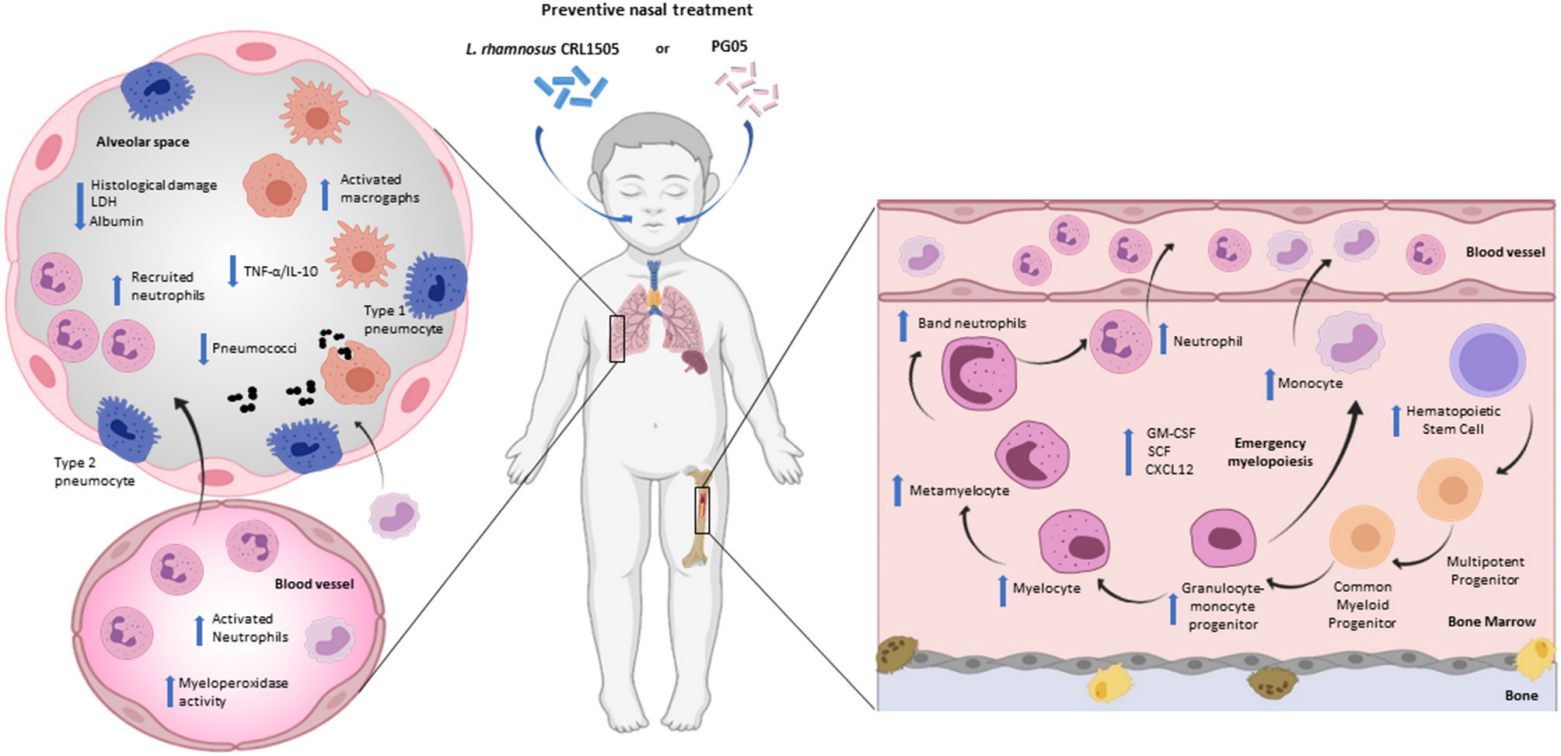
Modulation of respiratory innate immunity by immunobiotic or postbiotic nasal treatments in malnourished host. Proposed mechanism for the distal immunomodulation induced by the immunobiotic strain *Lactobacillus rhamnosus* CRL1505 or its peptidoglycan and the enhancement of the resistance against *Streptococcus pneumoniae* infection through the improvement of the lung innate immune response and myelopoiesis (Figure created with Biorender.com).

The nasal administration of *L. rhamnosus* CRL1505 during repletion diet induces increased resistance to infection by *S. pneumoniae* in mice (38, 52). This immunomodulatory effect is similar to that of PG05, the cellular wall or the non-viable form of CRL1505 strain (19). When we performed a comparative study the effect of PG05 and peptidoglycans from *L. plantarum* CRL1506 (immunomodulatory strain), and *L. rhamnosus* CRL534 (non-immunomodulatory strain), we demonstrated that PG05 has unique immunomodulatory properties that cannot be extended to peptidoglycans from other strains (20). The increase in resistance to infection was evidenced by the fast elimination of *S. pneumoniae* from the lungs, the reduction of lung damage demonstrated by the decrease in LDH activity and the concentration of albumin in bronchoalveolar lavage, accompanied by preserved histological characteristics of the lungs (19, 20, 36) (Figure 2). Besides bacterial infections, nasal probiotics have also been used for viral respiratory infections in mice caused by Influenza virus (53–56), Respiratory Syncytial Virus (13) and Pneumovirus (57).

During the innate immune response against *S. pneumoniae*, protein-deprived malnourished mice that received preventive intranasal administration of the strain CRL1505 or PG05, show an increase in leukocyte counts at the respiratory level, accompanied by a recruitment of neutrophils at the alveolar level (CD45^+^Gr1^+^ cells) (19, 38). Unlike the viable strain that induces an increase in the number of leukocytes and neutrophils in peripheral blood, PG05 does not modify these parameters. However, the functionality of neutrophils has been increased by the immunobiotics and the postbiotics (19, 38). These findings could be related to the ability of these treatments to enhance emergency myelopoiesis, since the CRL1505 strain is capable of promoting the proliferation and differentiation of myeloid progenitors and increasing growth factors such as GM-CSF and G-CSF in bone marrow (38). In addition, the preventive nasal treatments with immunobiotic can modulate the CXCR4/CXCL12 signaling axis that regulates the exit of myeloid cells during emergency myelopoiesis. Consequently, preventive treatment with PG05 induces an increase in the number of alveolar macrophages (CD11c^+^F4/80^+^MHCII^+^ cells) and MHCII expression in both lung and spleen macrophages (19, 20) (Figure 2).

Namely, immunobiotics can influence blood IL levels (18), which is consistent with our findings that demonstrate the ability of *L. rhamnosus* CRL1505 and its peptidoglycan to normalize serum levels of TNF-α, IL-1β, and IL-6 in protein-deprived malnourished mice during innate immune response against *S. pneumoniae*. Furthermore, these preventive treatments increase the serum levels of IL-10 and INF-γ (19, 38). Despite the fact that the challenge with *S. pneumoniae* induces an increase in the levels of pro-inflammatory cytokines and chemokines at the pulmonary level, the supplementation of the diet with *L. rhamnosus* or PG05 shows a low TNF-α/IL-10 ratio. This modulation of the inflammatory response has a direct relationship with recovery of lung histopathology (19, 20, 38). We speculate that other innate response cytokines and chemokines, such as KC or MCP-1 (58, 59), produced by pulmonary epithelial cells or alveolar macrophages, could be involved in modulation of the inflammatory response. Moreover, this cytokine balance induces by *L. rhamnosus* or its PG05 could be responsible for the increase in the number of phagocytic cells in the lung, but also in the activity of blood peroxidase and the number of macrophages in the spleen. In this way, new questions are opened about the mechanisms that underlie the capacity of immunobiotics and postbiotics on myelopoiesis in malnutrition.

## Protein-Malnutrition Impairs the Respiratory Specific Immune Response Against Pneumococcal Infection

To achieve complete protection against pneumococcus, both innate and adaptive immune mechanisms are necessary (60). Pneumococcal exposure leads to the generation of both T-cell and B-cell immune responses to polysaccharide and protein antigens (61, 62). However, malnutrition affects the lymphopoietic organs, altering the immune response. It is widely accepted that nutritional deprivation leads to lymphoid atrophy, as demonstrated in animal models (63, 64) and clinical trials (65, 66). Cellular apoptosis plays a key role in altering lymphopoiesis and atrophy of lymphoid tissue, as thymus and spleen, during malnutrition by protein deprivation (67). Furthermore, nutritional deficiencies increase apoptosis in peripheral blood lymphocytes of malnourished children (68).

The thymus is the main organ of thymopoiesis and a key target organ in malnutrition. Taking into account that T cells are a main component of the specific immune response, several research have described the effect of protein deprivation on the number and function of T cells. In clinical studies in malnourished individuals, spoilage of T cell functionality (66), decrease in the number of CD4 and CD8 T cells in the blood (69) and deterioration of thymic T cell production (65–67) was observed. On the other hand, animals fed a protein-deficient diet showed thymic atrophy, reduced number of thymic cells and lymphocytes (70), and decreased number of T cells (71, 72). Even more, Barbieri et al. (70) demonstrated a reduced entry into the thymus of CD4^−^CD8^−^ double negative cells (DN) from bone marrow in protein-deprived malnourished mice. In thymus, malnutrition affects the four stages of maturation according to the differential expression of CD44 and CD25: CD44^+^CD25^−^ (DN1), CD44^+^CD25^+^ (DN2), CD44^−^CD25^+^ (DN3) and CD44^−^CD25^−^ (DN4) (70). Consequently, both CD4^+^CD8^+^ double positive (DP) CD3^−^/^low^αβTCR^low^ thymocytes, more abundant cells of the thymus that are derived from DN4 thymocytes, as well as CD4^+^ and CD8^+^ simple positive (SP) CD3^high^TCR^high^ immunocompetent T cells that leave the thymus toward the periphery decrease significantly in protein-deprived malnourished mice (70, 73). Although nutritional deprivation induces a reduction in the number of T lymphocytes and CD8^+^ cells in bone marrow, spleen and lung, an increase in CD4^+^ T cells is observed in bone marrow. This increase is accompanied by a decrease in CD4^+^ T cells in the spleen and lung. It is possible that the bone marrow tries to restore normal hematopoiesis in the face of hypoplasia-induced stress present in protein malnutrition (63, 74, 75). In the protection against *S. pneumoniae*, the induction and maintenance of antigen specific T cell responses is essential. However, malnourished animals are unable to effectively increase the levels of certain cytokines, such as IL-2, IL-4, INF-γ, or IL-10, in response to *S. pneumoniae* at the respiratory or systemic level (20, 70).

As mentioned above, protein malnutrition induces a significant reduction in the cellular compartments of the bone marrow (17), and this has been shown to be negatively affected in the B cell population (63). In adults, B cells are generated in the bone marrow, reaching the stage of transitional B cells, which are short-lived and functionally immature. These cells develop into mature B cells in the spleen and recirculate between the lymph nodes (76). In this sense, it is known that nutritional deprivation impairs the populations of B cells in the bone marrow, observing a reduction of the complete B cell compartment (B220^+^ cells) in malnourished mice (36, 63). In parallel with the total decrease of B cells, it is observed that the number of pro-B/pre-B (B220^interm^IgM^−^) and immature B cells (B220^interm^IgM^+^) is lower in the mice deprived of protein. This reduction in immature B cells is accompanied by an increase in the percentage of mature B cells (B220^high^IgM^+^) but not by changes in the total number of mature B cells (63). Furthermore, malnutrition reduces the number of spleen B cells as well as all subpopulations of B cells, such as mature (CD19^+^B220^High^CD24^Low^IgM^+^), immature (CD19^+^B220^Low^CD24^High^ IgM^+^), transitional 1 (IgD^+^IgM^High^CD24^High^) and transitional 2 (IgD^−^IgM^High^CD24^High^) B cells (36). These observations suggest that nutritional deprivation of protein leads to impaired development of B cells in the bone marrow and spleen.

Mature B cells play an important role in the specific immune response by producing antibodies after being stimulated, expanded and selected in the germinal centers in the presence of the help of T cells (76). During pneumococcal infection the number of spleen lymphocytes and total B cells increases (CD19^+^B220^+^ cells). However, protein-deprived malnourished mice show a much smaller increase than normal controls (36). A detailed study of lung B cell subpopulations shows that infection reduces the number of lung lymphocytes, without affecting the number of CD19^+^B220^+^ cells. Also, mature B cells decrease after infection, while immature B cells increase. However, malnourished mice have fewer of these subpopulations (36). The number and activity of B and T cells have been reported to be related to the impairment of the humoral immune response in malnourished children (77). It has been shown that protein malnutrition markedly reduces bronchoalveolar lavage and serum anti-pneumococcal antibodies (36). Furthermore, the opsonophagocytic activity of IgG antibodies was significantly reduced in malnourished mice (36). These findings are associated with the deterioration of the B cell population in the bone marrow but without affecting its ability to produce antibodies (36, 63).

## Immunobiotics and Postbiotics Accelerate the Respiratory Specific Immunity Recovery of Malnourished Hosts

The recovery of T cells is important for host protection through the production of cytokines that control and coordinate several immune effector mechanisms and their ability to influence antibody production by B cells.

The active thymopoiesis is characterized by high CD3 expression and increased frequency of DP T cells (78). In the absence of infection, the renutrition treatments supplemented nasally with *L. rhamnosus* CRL1505 and its PG05 to protein-deprived malnourished animals is capable of activating thymopoiesis by increasing the number of DP T cells (20, 70). These treatments induce an increase in mature T cells, with a more remarkable effect on the CD4 SP population. Consequently, this nasal treatment increases the number of CD3^+^CD4^+^ cells in spleen and lungs. Thus, the administration of lactobacilli during a renutrition diet could induce an accelerated development of CD4 SP T cells in the thymus and their migration to peripheral tissues (20, 70).

**Figure 3:**
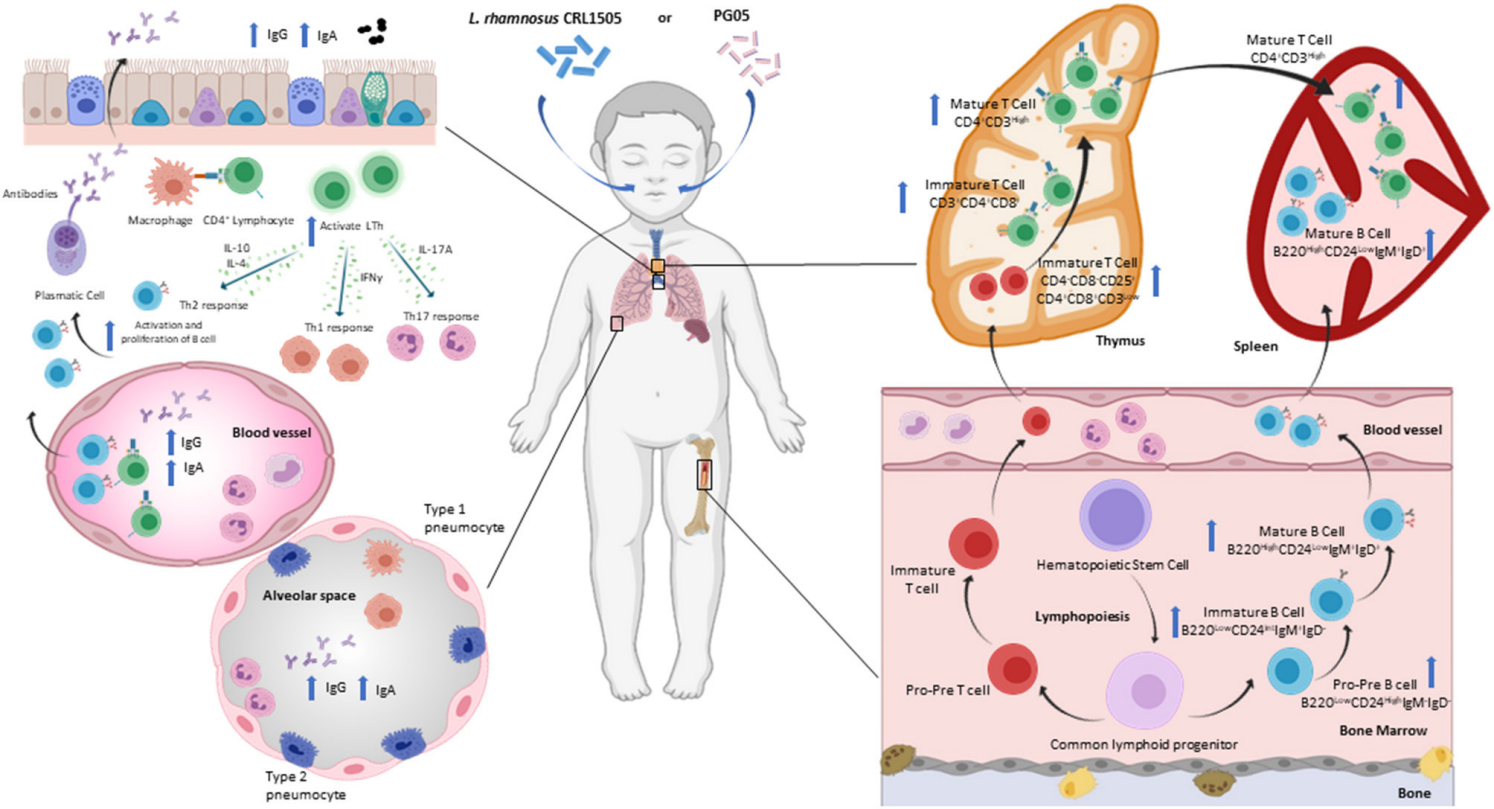
Modulation of respiratory specific immunity by immunobiotic or postbiotic nasal treatments in malnourished host. Proposed mechanism for the distal immunomodulation induced by the immunobiotic strain *Lactobacillus rhamnosus* CRL1505 or its peptidoglycan on the resistance against *Streptococcus pneumoniae* infection, maturation and differentiation of T and B cells in bone marrow, spleen, thymus and lung (Figure created with Biorender.com).

As mentioned above, T cell mediated immune responses are important in the defense against *S. pneumoniae*. The supplementation of a renutrition diet with nasal administration of *L. rhamnosus* CRL1505 and its PG05 promotes the increase of DN T cells, at the expense of the maturation stages DN1 and DN4 (CD25^−^DN thymocytes) (20, 70). Taking into account that DN1 cells correspond to progenitors from bone marrow (73, 79) and that immunobiotic treatments accelerate the recovery of hematopoiesis in protein-deprived malnourished animals (44, 63), the increase in CD25^−^ DN thymocytes is likely due to the increased influx of T precursor from the bone marrow to the thymus during pneumococcal infection. In addition to promoting the development of CD4^+^ T cells in the thymus after the pneumococcal challenge, the treatment with *L. rhamnosus* CRL1505 managed to normalize the spleen and bone marrow CD3^+^CD4^+^ population, and maintain the values of lung CD3^+^CD4^+^ cells higher than the malnourished mice (70). This effect of *L. rhamnosus* CRL1505 is similar to that induced by PG05 (20), and could be key for protection against *S. pneumoniae* infection (80) (Figure 3).

A pneumococcal infection triggers a Th2 response and cytokines in the airway environment change dramatically. During this response, the production of IL-4, IL-5, IL-6 and IL-10 increases, which helps to stimulate B cells to proliferate and mature into cells that produce anti-pneumococcal IgG, IgM and IgA antibodies (81). B cell antibody class switching depends essentially on the Th2 cells (82, 83). Nasal administration of *L. rhamnosus* CRL1505 or its PG05 during renutrition induces a positive regulation of the Th2-cytokine IL4 at the local and systemic level (20, 70). This modulation is coherent with the increase in anti-pneumococcal antibodies found after the application of nasal treatments in malnourished mice. Barbieri et al. (36) demonstrated the increase in anti-pneumococcal IgA and IgG at the serum and respiratory levels due to immunobiotic modulation, while Kolling et al. (20) showed the highest local and systemic anti-pneumococcal antibody production (IgG, IgM and IgA) by stimulation with specific postbiotics. These findings are in agreement with previous reports (83, 84). IL-10 is known to modulate the immune response induced after a pneumococcal infection, limiting the inflammatory immune response and stimulating antibody production in both children and mice (85, 86). In this sense, nasal administration of immunobiotics or PG05 is responsible for increasing IL-10 levels in bronchoalveolar lavage of malnourished mice after pneumococcal infection (20, 70). This increase is critical to reduce tissue damage and neutrophil recruitment to the airways. Furthermore, only PG05 induces the production of IL-2, which is involved in the CD4^+^ T cell response against pneumococcal antigens (87). On the other hand, the role of the Th1-cytokine IFN-γ in protection against pneumococcal infection is very complex. Low levels of IL-10 are known to be associated with high levels of INF-γ during pneumococcal infection, leading to exacerbated inflammation (86). This imbalance, which is characteristic of protein-deprived malnutrition, is reversed in malnourished animals that received nasal treatments during renutrition, and is associated with their greater ability to eliminate the pathogen from the lungs (20, 36). Nasal administration of immunobiotics and postbiotics represents a non-invasive means to modulate and enhance T-cell-mediated immunity against respiratory pathogens in immunosuppressed malnourished hosts. Further studies are needed to elucidate the type of CD4^+^ T cells involved in enhancing defense against pneumococcal infection.

The humoral response against *S. pneumoniae* in the upper respiratory tract results in the production of IgA that can protect the host against pathogen colonization (88). In the alveolar space, *S. pneumoniae* induces the differentiation and expansion of plasma cells secreting IgG antibodies (89, 90). IgG antibodies are opsonizing, allow complement fixation, and enhance the microbicidal activity of macrophages. Humoral immune activation in the lungs also induces the systemic antibodies production to prevent the passage of *S. pneumoniae* into the blood (91). As previously stated, protein-deprivation impairs the production of mucosal IgA and IgG (36, 92) and opsonophagocitic activity of IgG antibodies in serum and bronchoalveolar lavage (36). Nasal preventive treatment with *L. rhamnosus* or its postbiotic induces an efficient humoral immune response with serum and respiratory IgG and IgA levels higher than malnutrition and well-nourished controls, as well as a greater opsonophagocytic activity (20, 36). In addition, the findings on improved antibody production are associated with an increase in mature and immature lung and bone marrow B cells and spleen mature B lymphocytes found in animals receiving preventive nasal treatments (20, 36) (Figure 3). Hence, the renutrition treatment supplemented nasally with *L. rhamnosus* CRL1505 or PG05 is able to increase the number and functionality of respiratory B cells. These treatments have the ability to impact bronchus associated lymphoid tissue and/or naso-pharynx-associated lymphoid tissue, and from there accelerate the recovery of central lymphoid sites such as bone marrow, thymus and spleen affected by malnutrition.

## Final Reflections

To date, no other nasal treatments in immunocompromised hosts are known to be as effective as those published by our group. Most of the known immunobiotics that can improve resistance to infection in immunocompromised hosts are administered orally (93, 94). However, our experience indicates that nasal administration is the optimal way to modulate the local response against infection with respiratory pathogens. The intestinal microbiota maintains the immune defense mechanisms in the respiratory tract, allowing efficient effector responses to the challenge of pathogens. Immune cells are directly exposed to bacterial products released in the intestine (41, 95). In addition to this gut-lung axis, the lung microbiota contributes significantly to airway tolerance and immune responses to respiratory infections (96, 97). Knowing the microorganisms that colonize the lungs in healthy and disease hosts, the metabolites they generate, and the immune response they modulate is the objective of current lines of research in the world (98). In this sense, immunobiotics or postbiotics, administered nasally, could act as intermediaries to modulate the immune responses triggered by PAMPs of respiratory pathogens. In this way, they could contribute to restoring and maintaining pulmonary homeostasis. These hypotheses open an interesting topic for future research.

In conclusion, the renutrition treatment supplemented nasally with *L. rhamnosus* CRL1505 or PG05 is able to accelerate the improvement of the number and functionality of respiratory myeloid, B and T cells. These treatments have the ability to impact the respiratory mucosa-associated lymphoid tissue, and from there accelerate the recovery of bone marrow, thymus and spleen damaged by malnutrition. Consequently, nasal supplementation with this immunobiotic or postbiotic results in an increase in resistance to a respiratory infectious challenge and an effective innate and specific immune response against the pathogen. The properties described for *L. rhamnosus* CRL1505 and its peptidoglycan, a new bioactive agent, underpin the scientific basis for their application as mucosal adjuvants in health plans, mainly aimed at improving the immune response of immunocompromised hosts. The search for new vaccine adjuvants that increase their effectiveness at the mucosal level is a problem of great scientific relevance today.

## Author Contributions

SS and SA wrote and revised the manuscript. All authors contributed to the article and approved the submitted version.

## Conflict of Interest

The authors declare that the research was conducted in the absence of any commercial or financial relationships that could be construed as a potential conflict of interest.

## Publisher's Note

All claims expressed in this article are solely those of the authors and do not necessarily represent those of their affiliated organizations, or those of the publisher, the editors and the reviewers. Any product that may be evaluated in this article, or claim that may be made by its manufacturer, is not guaranteed or endorsed by the publisher.
